# The Relationship Between Trait Emotional Intelligence and Personality. Is Trait EI Really Anchored Within the Big Five, Big Two and Big One Frameworks?

**DOI:** 10.3389/fpsyg.2019.00866

**Published:** 2019-04-24

**Authors:** Alberto Alegre, Núria Pérez-Escoda, Elia López-Cassá

**Affiliations:** ^1^Department of Early Childhood and Elementary Education, East Stroudsburg University, East Stroudsburg, PA, United States; ^2^Department of Research Methods and Diagnosis in Education, University of Barcelona, Barcelona, Spain

**Keywords:** personality, five factor model, big 5, big 2, stability, plasticity, general factor of personality, trait emotional intelligence

## Abstract

Pérez-González and Sánchez-Ruiz (2014) published a study in which they found that trait emotional intelligence can be considered a broad personality trait integrated into the higher levels of a multi-level personality hierarchy. They also came to the conclusion that this construct can be considered a proxy for the general factor of personality. The purpose of this study is to try to replicate their study. We follow the same methodology these authors used but with a new sample, and a different definition of trait emotional intelligence and therefore a different measurement tool. Our results show convergent validity between trait emotional intelligence and personality, but not discriminant validity, suggesting than trait emotional intelligence is not integrated in the higher level of the personality hierarchies, but it is another way to measure the same big five personality traits that traditionally compose the construct of personality. We also found that trait emotional intelligence highly correlated with the general personality factor, but additionally we found an extremely high negative correlation between those two constructs and neuroticism. This finding suggest that they may represent above all just the absence of neuroticism in a person.

## Introduction

Personality is traditionally studied in terms of human traits, and the most popular classification of personality traits is the well know Big Five (B5) or Five Factor Model (Goldberg, 1981; Costa and McCrae, 1987, 1992). About 20 years ago, because of the use of statistical factor analysis, those B5 traits were grouped in two bigger personality factors (B2; Digman, 1997): one that Digman termed Alpha and that DeYoung et al. (2002) renamed as Stability encompasses the personality traits of agreeableness -A, conscientiousness -C, and neuroticism -N. The second factor that Digman called Beta and DeYoung et al. (2002) termed Plasticity encompasses extroversion –E- and openness -O-. De Young and colleagues have been able to find support for the existence of these two-factors in different studies (DeYoung et al., 2002, 2007), and so has done Saucier (2010). Additionally, the model replicates reliably across cultures and languages (Saucier et al., 2014).

Further factor analyses revealed, in other studies, the existence of one general factor of personality (Rushton and Irwing, 2008; van der Linden et al., 2010). Rushton et al. (2008) proposed that this general personality factor (GFP) has evolved over centuries as a result of natural selection. According to this theory, it would be transferred via genetic inheritance, largely reflecting social effectiveness (Rushton, et al., 2008), and would represent an evolutionary adaptive trait with a survival advantage, especially in social situations, to any other personality trait or combination of traits (Figueredo and Rushton, 2009). Research shows that the GFP does in fact transfer genetically (van der Linden et al., 2017), and correlates to an array of positive social outcomes (see van der Linden et al., 2017 for a review). Some authors have argued that the GFP is nothing more than a statistical artifact caused by individuals’ tendency to give socially desirable responses. However, van der Linden et al. (2016) showed that while some degree of bias can be expected, the GFP most likely is a real construct.

Another construct has been proposed in the last years as providing an advantage in social situations. It is called emotional intelligence (Mayer et al., 1991). Different authors such as Goleman (1995) and Brackett et al. (2006) directly or indirectly suggest that this construct would be adaptive because it provides abilities particularly useful in situations where an effective way of managing emotions and relationships is more important than the use of brute force in order to achieve goals. This proposition is supported by findings showing that the ability of living creatures to experience emotions allows the organism to respond more adequately to threats in the environment, thus providing an evolutionary advantage (Nesse, 1990; de Waal, 2011). Moreover, Fischman (1993) showed that evolution has favored individuals with the ability to better regulate their brain activity and therefore their thoughts and emotions. Furthermore, Kret et al. (2018) proposed that the ability to recognize emotions in others and adequately yet quickly responding to them is crucial for survival.

### Trait Emotional Intelligence

From the very beginning, there has been strong controversy about the definition and nature of emotional intelligence. Lately, there seems to be some agreement among experts that there are two types of emotional intelligence: one termed ability emotional intelligence, which entails a particularly high ability to process emotional information and that is related to, but distinct from cognitive ability. The second, termed trait emotional intelligence (trait EI), is a construct first proposed by Petrides and Furnham (2001). It relates to personality, and represents a combination of personality traits, particularly effective in situations with emotional and social implications.

Over the years, different authors have provided ample evidence of the existence of trait EI, finding relationships with happiness (Petrides and Furnham, 2003; Ye et al., 2018), self-esteem (Ziasma et al., 2015), loneliness (Zou, 2014), and job satisfaction (Platsidou, 2010) among many other positive outcomes.

Moreover, because, trait EI is predicated to be a personality trait including a constellation of emotion-related dispositions and self-perceptions (Petrides et al., 2007b), it was necessary to show that it correlates with other personality measures. Indeed, research findings have shown relationships between trait emotional intelligence and the B5, and especially between Neuroticism and Extroversion and trait EI (Saklofske et al., 2003; Vernon et al., 2008; Siegling et al., 2015). It also was important to understand which place trait EI occupied within established personality hierarchies to demonstrate its discriminant validity. Petrides et al. (2007b) were able to show that when factor analyzed in conjunction with other personality scales, trait EI appeared as a differentiated factor.

To consider trait EI a valid and important construct, nevertheless, it was also necessary to show that it had the ability to predict outcomes beyond those predicted by other personality constructs. Petrides et al. (2007b) again were able to show the incremental ability of trait EI to predict life satisfaction, rumination, and coping strategies.

In addition, from their own definitions, it made sense to think that the general factor of personality and the construct of trait EI would show a strong overlap. Both are composites of personality traits and both are supposed to be effective in social endeavors. In order to test this possibility further, Pérez-González and Sánchez-Ruiz (2014), in a study with 289 university students, investigated the relation between trait emotional intelligence and the B5 personality traits, the B2, and the Big 1 or General Factor of Personality. They found that, as expected and as found in previous studies (Petrides et al., 2007a, 2010), all five personality traits correlated with trait emotional intelligence. Moreover, using regression analysis, they were able to show that four of the B5 traits were significant predictors of trait EI (all except Agreeableness), and they jointly predicted 57.3% of trait EI variance. Because of those two results, trait EI demonstrated, according to the authors, convergent validity with the B5. Additionally, using exploratory factor analysis, they were able to show that trait EI emerged as a distinct oblique factor under the B5 factor space. That would mean, again according to these authors, that trait EI showed also discriminant validity with respect to the B5.

In terms of the B2, stability is hypothesized to contribute mainly to social and emotional adjustment. Plasticity, on the other hand, would be a facilitator of social learning (Pérez-González and Sánchez-Ruiz, 2014). Those two factors, plasticity and stability have been replicated in several studies (Rushton and Irwing, 2008; Şimşek, 2014; Liu and Campbell, 2017). Pérez-González and Sánchez-Ruiz suggested that trait EI would be more strongly related to Stability. In their study, they were able to confirm this hypothesis. Those results again indicated convergent validity and consistency with the hypothesis previously explained.

Finally, in regards to the general factor of personality (GFP), Pérez-González and Sánchez-Ruiz found a strong correlation between trait EI and the GFP. They also found that the ability of the B5 to predict trait EI was mostly due to those traits included in the GFP. In fact, the GFP correlated more strongly with trait EI than with any of the B5 traits. With all these results, Pérez-González and Sánchez-Ruiz concluded that trait emotional intelligence not only relates to the general factor of personality, but it is a broad personality trait integrated into multi-level personality hierarchies. They believed that trait emotional intelligence could be considered as a proxy of the General Factor of Personality.

The purpose of this study is to replicate Pérez-Gonzalez and Sánchez Ruiz’s study using a new measure of trait EI developed by Pérez-Escoda et al. (2010a), and obtain results that either support or do not support their conclusions. Additionally, the idea of the existence of a superior type of personality that would be more efficient than any other in social situations is a bold proposition and one that definitely needs more research support than it can show as of now. If we can confirm that trait EI can be considered a proxy for GFP, or in other words, that they are basically the same, we would provide support for this thesis. Trait EI has shown already that it is an advantage in social situations (Brackett et al., 2006). Showing that trait EI and the GFP are the same thing would mean that the GFP is also an advantage in social situations, and that would provide support for the thesis that the GFP is a real type of personality that has consolidated over time because it provides an evolutionary advantage.

## Materials and Methods

### Participants

The sample consisted of 497 Spanish university students (86% female) ranging in age from 19 to 64 years old (*M* = 29.39, *SD* = 9.28), and with a level of studies ranging from elementary schooling to doctorates and a mode of undergraduate university studies.

### Measures

#### B5

We measured the B5 factors using a personality questionnaire known as NEO-FFI (Costa and McCrae, 1992), which is a reduced version of the longer NEO Personality Inventory-Revised (NEO PI-R; Costa and McCrae, 1989). The NEO-FFI has demonstrated, according to its authors, high Cronbach reliability coefficients for all scales: Neuroticism (0.86), Extroversion (0.77), Openness (0.73) for, Agreeableness (0.68), and Conscientiousness (0.81). We report the internal consistencies obtained in the current study in parentheses in Table 1. The test consists of 60 items and is aimed at young adults and adults with a minimum level of instruction of sixth grade. It can be administered individually or in groups. It uses a Likert type scale with five response options that go from totally disagree to totally agree. We used the Spanish version of the NEO-FFI that has been translated and validated by TEA Ediciones (Cordero et al., 2008).

**Table 1:** Inter-correlations among TEI, the B5, the B2, and the GFP.

	GPF	Stability	Plasticity	O	C	E	A	*N*	Total EI
GPF (0.88)	1								
Stability (0.87)	0.783**	1							
Plasticity (0.82)	0.423**	0.189**	1						
Openness (0.77)	0.092*	0.083	0.786**	1					
Conscientiousness (0.80)	0.675**	0.838**	0.093*	0.015	1				
Extraversion (0.82)	0.573**	0.215**	0.791**	0.244**	0.130**	1			
Agreeableness (0.69)	0.511**	0.752**	0.209**	0.135**	0.276**	0.194**	1		
Neuroticism (0.86)	-0.855**	-0.532**	-0.200**	0.017	-0.417**	-0.330**	-0.343**	1	
Total emotional intelligence (0.89)	0.730**	0.521**	0.461**	0.257**	0.396**	0.468**	0.389**	-0.678**	1


*N = 497. GPF = General Personality Factor, O = Openness, C = Conscientiousness, E = Extraversion, A = Agreeableness, N = Neuroticism, Total EI = Emotional Intelligence total coefficient. ^∗∗^Correlation is significant at the 0.01 level (2-tailed), ^∗^Correlation is significant at the 0.05 level (2-tailed).*

#### Trait Emotional Intelligence

We measured Trait emotional intelligence with the Emotional Development Questionnaire for Adults (CDE-A35; Pérez-Escoda and Alegre, in press). This scale is based on Bisquerra and Pérez Escoda (2007) trait EI definition as the set of knowledge, skills, abilities and attitudes necessary to understand, express and regulate emotional phenomena appropriately. It consists of 35 items, rated on a 10-point Likert scale, and it covers five distinct dimensions, namely Emotional Awareness, Emotional Regulation, Emotional Autonomy, Social Competence, and Life and Well-being abilities. The scale also provides a total trait EI score. The CDE-A35 is particularly adapted to the Spanish language and culture, which made it the best choice for our sample.

Pérez-Escoda and Alegre validated this instrument with a sample of more than 3000 respondents. They were either graduate students or participants in diverse emotional competence workshops offered in different parts of Spain. The age of the participants ranged from 18 to 67 years with an average of 35.28 (*SD* = 11.27). In their study, they report good reliability for all the scales (Social skills = 0.66, Autonomy = 0.74, Life and well-being competencies = 0.82, Emotional awareness = 0.80, Emotional regulation = 0.78, and total trait EI = 0.92). They also report positive correlations with others measures of emotional intelligence or close constructs. For instance, the correlation with the constructive thinking coefficient or PGC measured with the Constructive Thinking Questionnaire (CTI; Cordero, 2001) was *r* = 0.82. The correlation with a social skills coefficient measured with the *Escala de habilidades sociales* (Social Skills Scale) (Gismero, 2000) was *r* = 0.66. Finally, the correlation with the emotional intelligence coefficient measured with the Spanish version of the Trait Meta-Mood Scale (Fernandez-Berrocal et al., 2004) was 0.39. Although this last correlation is not as high as the previous two, it is very similar to the correlation (*r* = 0.43) that Brackett and Mayer (2003) found between the SREIT (Schutte et al., 1998) and the EQ-I (Bar-On, 1997), two measures of trait emotional intelligence that are well established. Therefore, those results showed strong evidence of convergent validity. In addition, the measure showed predictive and incremental validity when the B5 personality traits, and the CDE-A35 coefficient were regressed on life satisfaction. Furthermore, a previous version of the same questionnaire also showed good reliability (Pérez-Escoda et al., 2010a), and validity (Pérez Escoda et al., 2010b). In the current study, the internal consistencies of the CDE-A35 scales were α = 0.63 for Social Skills, 0.80 for Life and Well-being competencies, 0.79 for Emotional regulation, 0.78 for Emotional Awareness, 0.77 for Autonomy and 0.90 for trait EI (global score).

#### Demographic Information

A brief questionnaire with only three questions collected information of the participants’ age, gender, and level of studies. The level of studies ranged from 1 = basic studies to 9 = Post-doctoral studies.

### Procedure

Students signed a consent form indicating their voluntary participation in the study. We collected the data during class time. Testing sessions lasted about a half hour.

## Results

### The Location of Trait EI in the B5 Factor Space

Moderate to high correlations were found between trait EI and the B5 (see Table 1); the highest correlation with Neuroticism (*r* = -0.68) and the lowest with Openness (*r* = 0.26). The mean inter-correlation between the global trait EI and the B5 was *r* = 0.44.

We carried out a multiple regression analysis with trait EI as the criterion variable and the B5 as predictors. We depict these results in Table 2 (see section “Introduction”). All the B5 traits were significant predictors of trait EI, and they jointly predicted 59.1% of its variance. All VIF values were below 1.5 showing that there were no multicollinearity issues.

**Table 2:** Multiple regression of trait EI onto the B5 and trait EI onto the B2.

				Standardized	
Section model		Unstandardized coefficients		coefficients	*t*
		B	Std. Error	Beta	
1	(Constant)	4.422	0.331		13.357
	Neuroticism	-0.065	0.004	-0.527^∗∗^	-15.323
	Extraversion	0.032	0.005	0.210^∗∗^	6.635
	Openness	0.030	0.005	0.198^∗∗^	6.600
	Agreeableness	0.021	0.006	0.108^∗∗^	3.453
	Concientiousness	0.018	0.005	0.116^∗∗^	3.627
	(Constant)	0.854	0.296		2.882
2	Stability	0.047	0.004	0.450^∗∗^	12.749
	Plasticity	0.036	0.003	0.376^∗∗^	10.645


*N = 497, ^∗∗^p < 0.01.*

We ran a principal axis factoring exploratory factor analysis of the five NEO-PI-R scales and the five CDE-A35 dimensions using Oblimin (delta = 0) rotation. Based on the examination of the Eigenvalues greater than 1, and the scree plot, the results offered a three-factor solution (see Table 3). As opposed to the results that reported Pérez-González and Sánchez-Ruiz, in our study, Trait EI did not emerge as a distinct oblique factor under the B5 factor space. On the contrary, different EI dimensions appeared associated with different personality factors. Life and well-being skills, autonomy, and social skills loaded in the first factor with neuroticism. Emotional awareness loaded in the second factor with extroversion. Agreeableness, conscientiousness, and emotional regulation loaded together in the third factor. Results were very similar using Promax (kappa = 4) rotation. In both analyses, the results indicate that emotional intelligence is not a distinct and unique factor within the B5 factor space, but rather that it represents a different way of measuring the same personality construct.

**Table 3:** Combined factor analysis of trait EI and B5 dimensions.

	Components		
	1	2	3
Competencies for life and wellbeing	0.801		
Neuroticism	-0.768		
Autonomy	0.767		
Social skills	0.762		
Extroversion	0.653		
Openness		0.813	
Emotional awareness		0.679	
Emotional regulation			0.786
Agreeableness			0.734
Conscientiousness			0.710


*Método de extracción: Análisis de componentes principales. Metodo de rotación: Normalización Oblimin con Kaiser.*

### Trait EI and the B2

We first investigated the existence of higher-order factors in the B5 latent space. The five NEO-FFI scales were again subjected to principal component analysis (PCA) using Oblimin (delta = 0) rotation (see Table 4) in which the Eigenvalues and the analysis of scree plot showed a two factor solution. Those two factors were clearly (eigenvalues > 1), equivalent to the Alpha/Stability (C, A, and N loadings) and Beta/Plasticity super-factors (O and E loadings) mentioned before. These two super-factors (B2) explained 60.48% of the variance, with Alpha/Stability explaining a higher percentage than Beta/Plasticity. We computed the Alpha and the Beta coefficients by adding the raw scores of all the items integrating each of the dimensions. Both coefficients correlated positively (*r* = 0.19, *p* < 0.01).

**Table 4:** EFA results showing factor loadings for the B2 and the GFP.

	B2		GFP	
	Stability	Plasticity	NEO-FFI	NEOFFI + TEI
Variance explained	-37.94	22.54	37.94	39.46
N	-0.822		0.775	0.778
C	0.749		0.657	0.513
A	0.636		0.666	0.511
O		0.852	0.259	0.285
E		0.687	0.595	0.576
Well-being and life skills				0.791
Emotional regulation				0.715
Autonomy				0.697
Social skills				0.679
Emotional awareness				0.562


*Extraction Method: Principal Component Analysis. Rotation Method: Promax with Kaiser Normalization.*

As observed in Table 1, the correlation between trait EI and Alpha/Stability (*r* = 0.52) was higher than the correlation between trait EI and Beta/Plasticity (*r* = 0.46). The B2 explained a substantial 41% of the variance in Trait EI, with Alpha/Stability as the strongest predictor (Table 2, see section “Materials and Methods”).

When we correlated the five trait EI competencies with Stability and Plasticity we found that Emotional Regulation, Life and Well-being competencies, and Autonomy related more strongly to Stability than to Plasticity, while Emotional Awareness and Social Skills correlated more strongly to Plasticity.

### Trait EI and the Big One

To identify the general personality factor, we retain one unique unrotated general component (GFP). The correlation between trait EI and the GFP was *r* = 0.73, which was higher than the correlations between the GFP and the B5 from which it was extracted except for Neuroticism. It was also a little higher than the correlation obtained by Pérez-González and Sánchez-Ruiz between those two variables (*r* = 0.69).

Following Van der Linden et al. (2012), we also performed a hierarchical regression analysis including trait EI as criterion, the GFP as predictor entered in step 1, and the individual B5 scales (O, C, E, A, and N) as predictors entered in step 2. Multicollinearity analysis showed there was a problem with Neuroticism and the Big 1. Therefore, we removed neuroticism from the regression equation and conducted a new analysis. The GFP in step 1 explained a substantial 53.2% of the variance in trait EI [R^2^_adj_:53.2, *F*(1,495) = 563.77, *p* < 0.001]. Concerning the total unique variance of the B5 scale scores in step 2, they explained an additional 5% of trait EI variance [R^2^_adj_:58, *F*(5,495) = 138.26, *p* < 0.001].

We conducted another single-factor PCA using Oblimin (delta = 0) rotation, whereby again the five NEO-FFI scales were combined together with the five CDE-A35 factors (see Table 4), but on this occasion we forced an unrotated one-factor solution. The loadings of the B5 on this factor ranged from 0.29 to 0.78, while those of trait EI ranged from 0.56 to 0.79. Similarly to Pérez-González results and others (see Rushton et al., 2009; Veselka et al., 2009a,b; McIntyre, 2010), the highest factor loadings on GFP corresponded to the five trait EI factors and N. However, in our case, E was also among the highest loadings, leaving C, A, and especially O as less important.

## Discussion

The goal of our study was to replicate the research carried out by Pérez-González and Sánchez-Ruiz (2014) on the relationship between trait emotional intelligence and the B5, the B2 and the B1 (or GFP) personality traits. In the aforementioned work, two types of evidence demonstrated the convergent validity of trait emotional intelligence. First, the five personality components correlated with trait EI, and second, each of the B5 personality traits predicted trait EI except for agreeableness, with a shared variance of 57%. In the current study, the B5 personality traits also correlated with trait EI, and they predicted it. In our case, even agreeableness contributed significantly in this prediction, and the B5 shared 63% of the variance with this variable. Therefore, we can say that our study confirms the convergent validity of the B5 in relation to trait EI.

Pérez-González and Sánchez-Ruiz (2014) based on a factorial analysis showed that the dimensions of trait EI emerged as an oblique and different factor from the five great factors of the personality. This result supported the thesis of discriminant validity between the B5 personality traits and trait EI. However, in our study, this separation of the dimensions of emotional intelligence in a factor other than the factors corresponding to the B5 did not occur. Quite the contrary, the factor analysis showed three factors, and all the emotional intelligence dimensions fell into different factors mixed with personality traits. These differences between the results seem to indicate that trait EI does not measure a different dimension of the personality, but measures the same personality construct determined by the B5. There may be two alternative explanations to this discrepancy. On the one hand, the difference between our results and those obtained by Pérez-González and Sánchez-Ruiz (2014) may be the consequence of our use of a different theoretical framework of trait emotional intelligence, and a different measuring instrument. Pérez-González and Sánchez-Ruiz (2014) based their study on the definition of emotional intelligence by Petrides and Furnham (2001), and used a measure of trait EI, the TEIQue, that offered four dimensions of emotional intelligence: well-being, emotionality, sociability and self-control. We based our study on the Bisquerra and Pérez Escoda, 2007 theoretical framework, and we used a measure of trait EI that offers five dimensions: emotional awareness, emotional regulation, social competence, emotional autonomy and competence for life and well-being. These data seem to indicate that the five emotional dimensions measured by the CDE-A35 coincide more strongly with the five major personality traits than the four dimensions measured by the TEIQue.

On the other hand, Oltmanns and Widiger (2018) found that when a large number of specific and highly correlated scales measuring a narrowly defined variant of one of the B5 domains are included in a factor analysis, they may separate from that the five main domains, appearing to be a separate construct. However, this separated factor is an artifact of the analysis, and in fact, it may just simply be part of that domain of the B5. This is what Cattell and Tsujioka (1964) called a “bloated specific factor.” That would be the case on Pérez-González’s study where trait EI was measured with many, probably highly correlated, scales. On the other hand, in our study where only a few scales measured trait EI, this bloated factor did not appear. New research should clarify if this is a problem of the construct of trait emotional intelligence, if it is a problem reduced exclusively to the way it is defined by Bisquerra and Pérez Escoda (2007), or if it is due to flaw on the statistics analysis performed by Pérez-González.

Another aspect of the study by Pérez-González and Sánchez-Ruiz (2014) was the identification of the B2 personality traits called Stability and Plasticity. Our study also identified these two broad traits, and as in the study by Pérez-González and Sánchez-Ruiz (2014), Stability was composed of the traits of Agreeableness, Conscientiousness and Neuroticism, while Plasticity grouped the traits of Extroversion and Openness. In our study, these two major traits also correlated with trait EI, and Stability was again the trait that shared the most variance with trait EI.

Pérez-González and Sánchez-Ruiz (2014) argued that trait EI and Stability have a greater correlation because both represent an intelligence of intrapersonal type, which in the case of the TEIQue rests in facets such as intrapersonal emotion regulation, stress management, self-esteem, and self-motivation. In the case of trait EI measured by the CDE-A35, the intrapersonal and interpersonal facets are combined in some of its dimensions. For instance, Emotional Awareness includes awareness of one’s own emotions as well as awareness of the emotions of others, and Competencies for Life and Well-being include the ability to adopt appropriate and responsible behaviors for the solution of personal problems, as well as satisfaction with oneself and with life. With the exception of Social Skills, which includes only interpersonal skills, in each of the other dimensions, those of an intrapersonal type, acquire a greater preponderance. For instance, Emotional Regulation includes awareness of the relationship between emotion, cognition and behavior, as well as the ability to self-generate emotions, and Autonomy includes facets such as self-esteem, positive attitude in life, responsibility, as well as personal self-efficacy all of which are of an intrapersonal nature.

Finally, in relation to the General Factor of Personality, the exploratory factor analysis also offered a single factor obtained without rotation, similar to that found by Pérez-González and Sánchez-Ruiz (2014). This GFP correlated strongly with trait EI confirming the findings of these authors. In fact, in our study, the correlation between these two variables was higher than that obtained by Pérez-González and Sánchez-Ruiz (2014). Additionally, similarly to their study, trait EI showed a correlation with the GFP higher than that shown by four of the B5 personality traits, a result that reinforces their thesis that trait EI can be considered a proxy of the GFP. In this sense, previous authors had proposed that trait EI would be the culmination of the GFP (Rushton and Irwing, 2011, p. 146), and the results mentioned would seem to confirm this hypothesis. However, the lack of discriminant validity between trait EI and the B5 introduces doubts about whether trait EI is a combination of personality traits at the highest level of the hierarchy of personality traits, or on the contrary, it is a new way of naming and grouping well known personality traits.

In addition, an unexpected result of our study is that the correlation between neuroticism and the GFP is so high (-0.86) that suggests that the GFP is basically the absence of neurotic tendencies. The high correlation between trait EI and neuroticism (-0.68) would seem to indicate that this phenomenon also applies to this construct. That is, based on our results, it could be interpreted that trait EI is indeed a proxy of the GFP, but that both more than a higher level of personality, represent an almost total absence of neurotic tendencies. These results resemble those obtained by other authors such as: Petrides and Furnham (2003); Freudenthaler et al. (2008), and van der Linden et al. (2017) who found very high correlations between trait EI and neuroticism (*r* = -0.73, of -0.76 and -0.58, respectively). van der Linden et al. (2018) also found neuroticism to load heavily on the GFP (-0.83), while the other four traits loaded much lower. Oltmanns et al. (2018) offer an explanation for this results. In a study with 1,630 adults from a community sample, they found very high correlations between the general factors of psychopathology (p factor), personality, (GFP), and personality disorder (g-PD). They concluded that the GFP overlap with the other two variables indicates that the GFP is an inverted measure of general life impairment. Based on this interpretation, it does make sense that in our study neuroticism, which is also a measure of some type of life impairment, shows a high correlation with the GFP.

On the other hand, and in apparent contradiction with the previous comment, among the dimensions of trait EI, the Life and Well-being competencies showed the greatest factorial weight. This is a very similar result to that of Pérez-González and Sánchez-Ruiz (2014) in whose study the dimension with the greatest factorial weight in the GFP was the so-called well-being. It seems that the competence of people to develop the behaviors and thoughts that allow them to experience well-being in their lives is at the core of a higher level of personality and of emotional intelligence. In a way, it seems to reinforce Maslow’s (1943) thesis that places self-realization at the top of the hierarchy of human needs, bringing the most satisfaction and well-being to the person. Moreover, this double finding raises the possibility that at the higher level of self-realization of the person is the ability to stop experiencing anxiety and anguish, and to enjoy the challenges and opportunities that every human being faces throughout of their life.

Pérez-González and Sánchez-Ruiz (2014) recommended in their article that future research explored the convergent and discriminant validity of emotional intelligence in relation to the personality traits proposed by different personality theories. This is a recommendation that we support. Nevertheless, we have done exactly the opposite in this study, that is, we have investigated whether an alternative model of trait emotional intelligence (in our case, the theoretical framework proposed by Bisquerra and Pérez Escoda, 2007) would present similar relationships with the same model of personality used by Pérez-González and Sánchez-Ruiz [and defined by Costa and McCrae (1992)]. It would be equally useful in the process of clarifying the relationships between emotional intelligence and personality to replicate this study using alternative definitions of both personality and emotional intelligence.

It should be noted that our study is not without limitations. In the first place, we obtained the information exclusively through questionnaires, all of them answered by the same respondents. This methodology tends to artificially increase the correlations between variables. However, in our case, the correlations are so high that they clearly outweigh the effect of this methodological limitation. In another aspect, the trait EI questionnaire used by Pérez-González and Sánchez-Ruiz (2014) measured 15 facets that were then grouped into four dimensions. This allowed them some analyses on the facets that were most related to the different groups of the most complete personality traits. In our case, the questionnaire measures only five dimensions of trait EI, but does not offer separate measures of the facets that make up each of these dimensions. Consequently, the analyses carried out in our study are more limited in their amplitude. In addition, the original study assessed the B5 with the NEO-PI-R and trait EI with the TEI-Que, while the present study assessed the B5 with the NEO-FFI (the short version of the NEO-PI-R that does not allow calculating facets) and trait EI with the CDE-A35. The use of different measurement tools was necessary because of time constraints, but it may have affected the results obtained.

In short, our study aimed to replicate and confirm the results obtained by Pérez-González and Sánchez-Ruiz (2014) that placed trait EI as a proxy of the general factor of personality. Our results confirm the findings in their study about the convergent validity of the B5 in relation to trait EI. However, they do not support their finding about the discriminant validity between the B5 and trait EI. On the contrary, our results indicate that trait EI is just another way to measure the same personality construct that the B5 measure. They confirm Pérez-González and Sánchez-Ruiz’s finding about the relation between trait EI and the B2, and show how much trait EI overlaps with stability. They also confirm their conclusion that trait EI can be considered a proxy of the GFP. Since trait EI is supposed to provide an advantage in our adaptation and survival, the GFP should be considered a combination of personality traits (or a type of personality) that also provide an adaptive advantage over other personality dimensions or combination of dimensions. However, our results point to the possibility that both the GFP and trait EI are mainly indicators of the absence of neuroticism in people and of their ability to enjoy life.

## Ethics Statement

This study was carried out in accordance with the recommendations of the University of Barcelona with written informed consent from all subjects. All subjects gave written informed consent in accordance with the Declaration of Helsinki. The protocol was approved by the University of Barcelona.

## Author Contributions

AA, NP-E, and EL-C have contributed equally to this manuscript. AA has developed the statistical analysis, the tables, and the conclusion. NP-E has developed the fieldwork, the collection of data, the processing of the data, and the review of the final version of the article. EL-C has developed the literature review, the search for references, the writing of the references, and the writing of the article.

## Conflict of Interest Statement

The authors declare that the research was conducted in the absence of any commercial or financial relationships that could be construed as a potential conflict of interest.

## References

[B1] Bar-On R. (1997). *The Emotional Quotient Inventory (EQ-I): Technical Manual.* Toronto: Multi-Health Systems, Inc.

[B2] Bisquerra R. Pérez Escoda N. (2007). Las competencias emocionales. *Educación XXI* 10 61–82.

[B3] Brackett M. A. Mayer J. D. (2003). Convergent, discriminant, and incremental validity of competing measures of emotional intelligence. *Pers. Soc. Psychol. Bull.* 29 1147–1158. 10.1177/0146167203254596 15189610

[B4] Brackett M. A. Rivers S. E. Shiffman S. Lerner N. Salovey P. (2006). Relating emotional abilities to social functioning: a comparison of self-report and performance measures of emotional intelligence. *J. Pers. Soc. Psychol.* 91 780–795. 10.1037/0022-3514.91.4.780 17014299

[B5] Cattell R. B. Tsujioka B. (1964). The importance of factor-trueness and validity, versus homogeneity and orthogonality, in test scales. *Educ. Psychol. Meas.* 24 3–30. 10.1177/001316446402400101

[B6] Cordero A. (2001). *CTI. Inventario de pensamiento constructivo. Una medida de la Inteligencia Emocional.* Madrid: TEA Ediciones.

[B7] Cordero A. Pamos A. Seisdedos N. (2008). *NEO PI-R, Inventario de Personalidad NEO Revisado. Adaptación española*.(3^a^ edición revisada y ampliada). Madrid: TEA Ediciones.

[B8] Costa P. T. Jr. McCrae R. R. (1987). Validation of the five factor model of personality accross instruments and observees. *J. Pers. Soc. Psychol.* 52 81–90. 10.1037//0022-3514.52.1.81 3820081

[B9] Costa P. T. Jr. McCrae R. R. (1989). *The NEO-PI/NEO-FFI Manual Supplement.* Odessa, FL: Psychological Assessment Resources.

[B10] Costa P. T. Jr. McCrae R. R. (1992). *Revised NEO Personality Inventory (NEO-PI-R) and NEO Five-Factor Inventory (NEO-FFI) Professional Manual.* Odessa, FL: Psychological Assessment Resources Inc.

[B11] de Waal F. B. M. (2011). What is an animal emotion? *Ann. N.Y. Acad. Sci.* 1224 191–206. 10.1111/j.1749-6632.2010.05912.x 21486301

[B12] DeYoung C. G. Hasher L. Djikic M. Criger B. Peterson J. B. (2007). Morning people are stable people: circadian rhythm and the higher-order factors of the big five. *Pers. Individ. Diff.* 43 267–276. 10.1016/j.paid.2006.11.030

[B13] DeYoung C. G. Peterson J. B. Higgins D. M. (2002). Higher-order factors of the B5 predict conformity: are there neuroses of health? *Pers. Individ. Diff.* 33 533–552. 10.1016/S0191-8869(01)00171-4

[B14] Digman J. M. (1997). Higher-order factors of the B5. *J. Pers. Soc. Psychol.* 73 1246–1256. 9418278 10.1037//0022-3514.73.6.1246

[B15] Fernandez-Berrocal P. Extremera N. Ramos N. (2004). Validity and reliability of the spanish modified version of the trait meta-mood scale. *Psychol. Rep.* 94 751–755. 10.2466/pr0 15217021

[B16] Figueredo A. J. Rushton J. P. (2009). Evidence for shared genetic dominance between the general factor of personality, mental and physical health, and life history traits. *Twin Res. Hum. Genet.* 12 555–563. 10.1375/twin.12.6.555 19943718

[B17] Fischman J. (1993). New clues surface about the making of the mind. *Science* 262 1517–1532. 8248800 10.1126/science.8248800

[B18] Freudenthaler H. H. Neubauer A. C. Gabler P. Scherl W. G. Rindermann H. (2008). Testing and validating the trait emotional intelligence questionnaire (TEIQue) in a German-speaking sample. *Pers. Individ. Diff.* 45 673–678. 10.1016/j.paid.2008.07.014

[B19] Gismero E. (2000). *EHS. Escala de Habilidades Sociales: manual*, 3d Edn Madrid: TEA Ediciones.

[B20] Goldberg L. R. (1981). Language and individual differences: the search for universals in personality lexicon. *J. Pers. Soc. Psychol.* 59 1216–1229.

[B21] Goleman D. (1995). *Emotional Intelligence: Why it Can Matter More Than IQ for Character, Health and Lifelong Achievement.* New York, NY: Bantam Books.

[B22] Kret M. E. Muramatsu A. Matsuzawa T. (2018). Emotion processing across and within species: a comparison between humans (*Homo sapiens*) and chimpanzees (*Pan troglodytes*). *J. Comp. Psychol.* 132 395–409. 10.1037/com0000108 30024235

[B23] Liu D. Campbell W. K. (2017). the big five personality traits, big two metatraits and social media: a meta-analysis. *J. Res. Pers.* 70 229–240. 10.1016/j.jrp.2017.08.004

[B24] Maslow A. H. (1943). A theory of human motivation. *Psychol. Rev.* 50:370.

[B25] Mayer J. D. Salovey P. Gomberg-Kaufman S. Blainey K. (1991). A broader conception of mood experience. *J. Pers. Soc. Psychol.* 60:100. 10.1037//0022-3514.60.1.100 1995832

[B26] McIntyre H. H. (2010). Gender differences in the nature and linkage of higher-order personality factors to trait and ability emotional intelligence. *Pers. Individ. Diff.* 48 617–622. 10.1016/j.paid.2009.12.019

[B27] Nesse R. M. (1990). Evolutionary explanations of emotions. *Hum. Nat.* 1 261–289. 10.1007/bf02733986 24222085

[B28] Oltmanns J. R. Smith G. T. Oltmanns T. F. Widiger T. A. (2018). General factors of psychopathology, personality, and personality disorder: across domain comparisons. *Clin. Psychol. Sci.* 6 581–589. 10.1177/2167702617750150 30221082 PMC6132273

[B29] Oltmanns J. R. Widiger T. A. (2018). Maladaptive variants of adaptive traits and bloated specific factors. *J. Res. Pers.* 76 177–185. 10.1016/j.jrp.2018.08.006 30906080 PMC6428437

[B30] Pérez-Escoda N. Alegre A. (in press). *Validación del Cuestionario. (de) Desarrollo Emocional de Adultos (CDE-A35)*.

[B31] Pérez-Escoda N. Bisquerra R. Filella G. Soldevila A. (2010a). Construcción del cuestionario de desarrollo emocional de adultos (QDE-A). *REOP* 21 367–379.

[B32] Pérez Escoda N. Filella G. Soldevila A. (2010b). Competencia emocional y habilidades sociales en estudiantes universitarios. *Revista Electrónica de Motivación y Emoción* 34 1–11.

[B33] Pérez-González J. C. Sánchez-Ruiz M. J. (2014). Trait emotional intelligence anchored within the big five, big two and big one frameworks. *Pers. Individ. Diff.* 65 53–58. 10.1016/j.paid.2014.01.021

[B34] Petrides K. V. Furnham A. (2001). Trait emotional intelligence: psychometric investigation with reference to established trait taxonomies. *Eur. J. Pers.* 15 425–448. 10.1002/per.416

[B35] Petrides K. V. Furnham A. (2003). Trait emotional intelligence: behavioural validation in two studies of emotion recognition and reactivity to mood induction. *Eur. J. Pers.* 17 39–57. 10.1002/per.466

[B36] Petrides K. V. Furnham A. Mavroveli S. (2007a). “Trait emotional intelligence: Moving forward in the field of EI,” in *The Science of Emotional Intelligence: Knowns and Unknowns (Series Inaffective Science)*, eds Matthews G. Zeidner M. Roberts R. (Oxford: Oxford University Press), 151–166. 10.1093/acprof

[B37] Petrides K. V. Pita R. Kokkinaki F. (2007b). The location of trait emotional intelligence in personality factor space. *Br. J. Psychol.* 98 273–289. 10.1348/000712606x120618 17456273

[B38] Petrides K. V. Vernon P. A. Schermer J. A. Ligthart L. Bloomsma D. I. Veselka L. (2010). Relationships between trait emotional intelligence and the B5 in the Netherlands. *Pers. Individ. Diff.* 48 906–910. 10.1016/j.paid.2010.02.019

[B39] Platsidou M. (2010). Trait emotional intelligence of greek special education teachers in relation to burnout and job satisfaction. *School Psychol. Int.* 31 60–76. 10.1177/0143034309360436

[B40] Rushton J. P. Bons T. A. Ando J. Hur Y. M. Irwing P. Vernon P. A. (2009). A general factor of personality from multitrait-multimethod data and cross-national twins. *Twin Res. Hum. Genet.* 12 356–365. 10.1375/twin.12.4.356 19653836

[B41] Rushton J. P. Bons T. A. Hur Y. M. (2008). The genetics and evolution of a general factor of personality. *J. Res. Pers.* 42 1173–1185. 10.1016/j.jrp.2009.01.005

[B42] Rushton J. P. Irwing P. (2008). A general factor of personality (GFP) from two meta-analyses of the Big Five: Digman (1997) and Mount, Barrick, Scullen, and Rounds (2005). *Pers. Individ. Diff.* 45 679–683. 10.1016/j.paid.2008.07.015

[B43] Rushton J. P. Irwing P. (2011). “The general factor of persoality: Normal and abnormal,” in *The Blackwell-Wiley Handbook of Individual Differences*, eds Chamorro-Premuzic T. Furnham A. von Stumm S. (New York, NY: Wiley), 132–161. 10.1002/9781444343120.ch5

[B44] Saklofske D. H. Austin E. J. Minski P. S. (2003). Factor structure and validity of a trait emotional intelligence measure. *Pers. Individ. Diff.* 34 707–721. 10.1016/S0191-8869(02)00056-9

[B45] Saucier G. (2010). The structure of social effects: personality as impact on others. *Eur. J. Pers.* 24 222–240. 10.1002/per.761

[B46] Saucier G. Thalmayer A. G. Payne D. L. Carlson R. Sanogo L. Ole-Kotikash L. (2014). A basic bivariate structure of personality attributes evident across nine languages. *J. Pers.* 82 1–14. 10.1111/jopy.12028 23301793

[B47] Schutte N. S. Malouff J. M. Hall L. E. Haggerty D. J. Cooper J. T. Golden C. J. (1998). Development and validation of a measure of emotional intelligence. *Pers. Individ. Diff.* 25 167–177.

[B48] Siegling A. B. Furnham A. Petrides K. V. (2015). Trait emotional intelligence and personality: gender-invariant linkages across different measures of the big five. *J. Psychoeduc. Assess.* 33 57–67. 10.1177/0734282914550385 25866439 PMC4361496

[B49] Şimşek ÖF. (2014). Higher order structure of personality and mental health: does general affectivity matter? *J. Pers. Assess.* 96 226–236. 10.1080/00223891.2013.836527 24066739

[B50] van der Linden D. Dunkel C. S. Petrides K. V. (2016). The general factor of personality as social effectiveness: review of the literature. *Pers. Individ. Diff.* 101 98–105. 10.1016/j.paid.2016.05.020

[B51] Van der Linden D. Schermer J. A. de Zeeuw E. Dunkel C. S. Pekaar K. A. Bakker A. B., (2018). Overlap between the general factor of personality and trait emotional intelligence: a genetic correlation study. *Behav. Genet.* 48 147–154. 10.1007/s10519-017-9885-8 29264815 PMC5846839

[B52] van der Linden D. Pekaar K. A. Bakker A. B. Schermer J. A. Vernon P. A. Dunkel C. S. (2017). Overlap between the general factor of personality and emotional intelligence: a meta-analysis. *Psychol. Bull.* 143:36. 10.1037/bul0000078 27841449

[B53] van der Linden D. te Nijenhuis J. Bakker A. B. (2010). The general factor of personality: a meta-analysis of big five intercorrelations and a criterion-related validity study. *J. Res. Pers.* 44 315–327. 10.1016/j.jrp.2010.03.003

[B54] Van der Linden D. Tsaousis I. Petrides K. V. (2012). Overlap between general factors of personality in the big five, giant three, and trait emotional intelligence. *Pers. Individ. Diff.* 53 175–179. 10.1037/a0025559 21967007

[B55] Vernon P. A. Villani V. C. Schermer J. A. Petrides K. V. (2008). Phenotypic and genetic associations between the big five and trait emotional intelligence. *Twin Res. Hum. Genet.* 11 524–530. 10.1375/twin.11.5.524 18828735

[B56] Veselka L. Schermer J. A. Petrides K. V. Cherkas L. F. Spector T. D. Vernon P. A. (2009a). A general factor of personality: evidence from the HEXACO model and a measure of trait emotional intelligence. *Twin Res. Hum. Genet.* 12 420–424. 10.1375/twin.12.5.420 19803769

[B57] Veselka L. Schermer J. A. Petrides K. V. Vernon P. A. (2009b). Evidence for a heritable general factor of personality in two studies. *Twin Res. Hum. Genet.* 12 254–260. 10.1375/twin.12.3.254 19456217

[B58] Ye J. Liu E. S. Rochelle T. L. (2018). Sequential mediating effects of provided and received social support on trait emotional intelligence and subjective happiness: a longitudinal examination in Hong Kong Chinese university students. *Int. J. Psychol.* 10.1002/ijop.12484 [Epub ahead of print]. 29611619

[B59] Ziasma H. K. Kausar L. ZaibUn N. Batool F. (2015). Emotional intelligence: a key factor for self esteem and neurotic behavior among adolescence of Karachi, Pakistan. *Indian J. Posit. Psychol.* 6 171–174.

[B60] Zou J. (2014). Associations between trait emotional intelligence and loneliness in Chinese undergraduate students: Mediating effects of self-esteem and social support. *Psychol. Rep.* 114 880–890. 10.2466/04.21.PR0.114k29w3 25074308

